# Transcriptomics Identifies Modules of Differentially Expressed Genes and Novel Cyclotides in *Viola pubescens*

**DOI:** 10.3389/fpls.2019.00156

**Published:** 2019-02-15

**Authors:** Anne L. Sternberger, Megan J. Bowman, Colin P. S. Kruse, Kevin L. Childs, Harvey E. Ballard, Sarah E. Wyatt

**Affiliations:** ^1^Department of Environmental and Plant Biology, Ohio University, Athens, OH, United States; ^2^Department of Plant Biology, Michigan State University, East Lansing, MI, United States; ^3^Interdisciplinary Molecular and Cellular Biology Program, Ohio University, Athens, OH, United States

**Keywords:** *Viola pubescens*, transcriptomics, gene co-expression analysis, cyclotides, genome assembly, mixed breeding, chasmogamous, cleistogamous

## Abstract

*Viola* is a large genus with worldwide distribution and many traits not currently exemplified in model plants including unique breeding systems and the production of cyclotides. Here we report *de novo* genome assembly and transcriptomic analyses of the non-model species *Viola pubescens* using short-read DNA sequencing data and RNA-Seq from eight diverse tissues. First, *V. pubescens* genome size was estimated through flow cytometry, resulting in an approximate haploid genome of 455 Mbp. Next, the draft *V. pubescens* genome was sequenced and assembled resulting in 264,035,065 read pairs and 161,038 contigs with an N50 length of 3,455 base pairs (bp). RNA-Seq data were then assembled into tissue-specific transcripts. Together, the DNA and transcript data generated 38,081 *ab initio* gene models which were functionally annotated based on homology to *Arabidopsis thaliana* genes and Pfam domains. Gene expression was visualized for each tissue via principal component analysis and hierarchical clustering, and gene co-expression analysis identified 20 modules of tissue-specific transcriptional networks. Some of these modules highlight genetic differences between chasmogamous and cleistogamous flowers and may provide insight into *V. pubescens’* mixed breeding system. Orthologous clustering with the proteomes of *A. thaliana* and *Populus trichocarpa* revealed 8,531 sequences unique to *V. pubescens*, including 81 novel cyclotide precursor sequences. Cyclotides are plant peptides characterized by a stable, cyclic cystine knot motif, making them strong candidates for drug scaffolding and protein engineering. Analysis of the RNA-Seq data for these cyclotide transcripts revealed diverse expression patterns both between transcripts and tissues. The diversity of these cyclotides was also highlighted in a maximum likelihood protein cladogram containing *V. pubescens* cyclotides and published cyclotide sequences from other Violaceae and Rubiaceae species. Collectively, this work provides the most comprehensive sequence resource for *Viola*, offers valuable transcriptomic insight into *V. pubescens*, and will facilitate future functional genomics research in *Viola* and other diverse plant groups.

## Introduction

**FIGURE 1:**
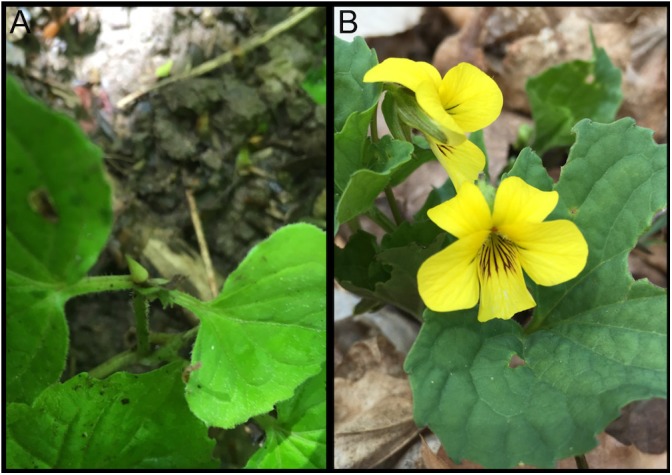
*Viola pubescens* var. scabriuscula bearing **(A)** cleistogamous and **(B)** chasmogamous flowers. Photographs were taken over native populations located in Sells Park, Athens County, Ohio, 45701 (39°20′40.6′′N 82°04′31.9′′W).

The genus *Viola* (violets) is distributed in both the northern and southern temperate regions as well as the tropics and possesses high diversity with 580–620 species, extensive allopolyploidy, and a distinct cytogenetic evolutionary history (Ballard et al., 1998; Marcussen et al., 2012, 2015; Wahlert et al., 2014). *Viola* is the largest genus in Violaceae, a family with moderately close relationships to the passionflower (Passifloraceae) and willow (Salicaceae) families in the order Malpighiales (Savolainen et al., 2000; Tokuoka and Hiroshi, 2006). Members of *Viola* exhibit frequent hybridization, diverse growth forms, assorted pollination and seed dispersal strategies, and varied breeding systems (Beattie, 1969, 1971; Beattie and Lyons, 1975; Ballard et al., 2011, 2014). Violets have been the fourth most popular bedding plant group (pansies), via sales, in the United States and abroad (Altland et al., 2003) and show potential for bioremediation (Hermann et al., 2013) and development of novel compounds for human use (Craik et al., 1999). *Viola pubescens* (Figure 1) is a perennial *Viola* herb commonly found in the understory of mesic forests in eastern North America. Most *Viola* species, including *V. pubescens*, possess and evolutionarily successful yet genetically uncharacterized mixed breeding system of both chasmogamous and cleistogamous flowers. While cleistogamous flowers are bud-like in appearance (Figure 1A) and mechanically sealed throughout their entire lifecycle, resulting in forced autogamy, chasmogamous flowers open at maturity, exposing their inner floral parts (Figure 1B). Cross-pollinated chasmogamous flowers have the advantage of sexual reproduction between two disparate parents offering genetically diverse progeny, reduced inbreeding depression, and removal of deleterious alleles from the population (Ballard et al., 2011). However, fertilization of chasmogamous flowers is contingent upon the availability of pollinating agents, and their nectar and showy floral organs require large amounts of energy and resources. The minute floral organs and lack of nectar in cleistogamous flowers make them less costly to produce and they have more resources for seed production including increases in overall seed number and/or larger seeds with higher viability (Culley and Klooster, 2007). Culley and Klooster (2007) conducted a survey investigating the occurrence of the chasmogamous/cleistogamous mixed breeding system, reporting a total of 536 species encompassing 41 diverse plant families, with the most occurrences reported in Poaceae (grasses), Fabaceae (legumes), Violaceae (violets), and Orchidaceae (orchids). Ballard et al. (2011) provided a comprehensive review of the literature on this mixed breeding system and highlighted the lack of information on the genetic basis of the system. However, the widespread distribution of the chasmogamous/cleistogamous mixed breeding system among monocot and dicot families as well as its expansive geographic range, suggests that the breeding system has evolved many times through the angiosperms (Ballard et al., 2011). This broad distribution also implies that the mixed breeding system is not a randomly occurring mating strategy and may be actively selected.

In addition to having evolutionarily advantageous mixed breeding systems, members of Violaceae also produce cyclotides. Cyclotides represent the largest circular protein family and have been classified as plant defense proteins based on their insecticidal (Jennings et al., 2001) and antimicrobial (Tam et al., 1999) properties but also have properties identified as anti-HIV (Gustafson et al., 1994), anti-cancer (Guzmán-Rodríguez et al., 2015), hemolytic (Tam et al., 1999), cytotoxic (Lindholm et al., 2002; Herrmann et al., 2008), trypsin inhibiting (Trabi and Craik, 2002) and uterotonic (Gran, 1973) among others (Zhang et al., 2009). Cyclotides are characterized by their cyclic cystine knot (CCK) motif of six conserved cys residues forming a tight network of disulfide bonds. This stable structure makes cyclotides resistant to proteolysis and strong candidates for drug design scaffolds and agrochemical applications (Craik et al., 1999; Gruber et al., 2008). With increased availability of genomic data, *in silico* methods have facilitated the discovery of many novel cyclotide sequences. The majority of cyclotides recently discovered are in Violaceae, which is speculated to contain upward of 30,000 unique cyclotides (Zhang et al., 2015). While only a small percentage of species in other cyclotide producing families have tested positive for cyclotide presence, cyclotide expression appears to be ubiquitous in Violaceae, and cyclotides have been identified in all species investigated (Burman et al., 2015; Göransson et al., 2015; Ravipati et al., 2017). While nine *Viola* transcriptomes have been sequenced to date (Supplementary Table S1), no *Viola* genome has been assembled (Matasci et al., 2014). The draft *V. pubescens* genome fills this gap in genomic data and provides a unique resource of sequencing and gene expression data. Here we present the *de novo* assembly and annotation of the *V. pubescens* draft genome and its use to investigate tissue-specific gene expression and cyclotide diversity in *V. pubescens*. These analyses provide insight into genetic disparities between chasmogamous and cleistogamous flowers and identified 81 cyclotide sequences.

## Results and Discussion

### Genome Size, Sequencing and Assembly

The genome size of *V. pubescens* was estimated through flow cytometry. The nuclear 2C DNA content was 0.93 pg with a standard deviation of 0.054 pg. Therefore, the haploid genome size of *V. pubescens* was estimated to be 455.7 ± 26.5 Mbp. For genome sequencing, genomic DNA obtained from leaf tissue of native *V. pubescens* plants was sequenced on an Illumina HiSeq 2000 (Illumina, Inc.) with paired-end, 100 bp chemistry. Two libraries were sequenced with estimated fragment sizes of 350–400 bp and 500 bp. Using the raw, trimmed DNA reads, the haploid genome size was also estimated via a k-mer distribution approach and was found to be ∼354 Mbp with 0.52% heterozygosity (Supplementary Figure S1 and Supplementary Table S2). Following adapter removal and filtering, 264,035,065 read pairs were assembled through ABySS (Simpson et al., 2009). Contigs were screened for contaminant reads using Taxon-Annotated GC-Coverage (TAGC) (Kumar et al., 2013; Laetsch and Blaxter, 2017), with no contamination detected. The assembly comprises 161,038 contigs covering 318 Mbp with an N50 of 3.45 kb and maximum scaffold length of 86.7 kb (Table 1). The majority of contigs were unscaffolded (96.8%) because of the limitations of fragmented, short-read sequencing data. The core eukaryotic genes mapping approach (CEGMA) was used to assess genome completeness. Out of the 248 core eukaryotic genes in CEGMA, 233 (94%) partial matches and 188 (76%) complete matches were found in the *V. pubescens* genome (Table 1). The Benchmarking Universal Single-Copy Orthologs (BUSCO) strategy was also used to evaluate genome completeness, and of the 2121 plant orthologs tested, 1691 (79.7%) complete matches were identified in the *V. pubescens* genome of which 287 (13.5%) were duplicated (Table 1). An additional 189 gene models (8.9%) were fragmented, and 241 (11.4%) were missing from the genome assembly. Repeats in the assembly were masked using RepeatMasker (v4.0.5) and default parameters. A custom repeat library was also generated but did not improve or substantially alter the default masking.

**Table 1:** Summary statistics of the *V. pubescens* genome assembly via ABySS and CEGMA and BUSCO assessments of genome completeness.

# of scaffolds	157,722
Total size of scaffolds (bp)	318,370,682
Longest scaffold (bp)	86,685
# of scaffolds > 1 K nt	80,885
N50 length (bp)	3,500
Average length of break (>25 N’s) between contigs in scaffold	45
Scaffold %N	0.06
Percent in scaffolded contigs	3.2
Percent in unscaffolded contigs	96.8
CEGMA Partial (%)	94, *n* = 248
CEGMA Complete (%)	76, *n* = 248
BUSCO	C: 79.7% [D:13.5%], F: 8.9%, M: 11.4%, *n* = 2121


*C: complete, D: duplicated, F: fragmented, M: missing, *n* = gene number.*

### RNA Sequencing and Transcriptome Assembly

RNA was extracted from basal stem, upper stem, petiole, leaf, peduncle, chasmogamous flowers, cleistogamous flowers, and fruit tissue of native *V. pubescens* populations. Three replicates of each tissue were sequenced using an Illumina HiSeq 2500 platform (Illumina, Inc.) and single-end, 50 bp chemistry. Post-cleaning and quality filtering, the number of reads per tissue ranged from 64.5 million reads in the leaf library to 126 million reads in the fruit library (Table 2). Transcripts were assembled *de novo* for each tissue type via Trinity, yielding a range of 22,363 transcripts in petioles to 37,183 in peduncles (Table 2). A *de novo* method was selected over a reference-based assembly due to the disparity between the genome and transcriptome sequencing depths.

**Table 2:** Number of RNA-Seq reads and transcripts identified across eight *V. pubescens* tissues.

Tissue	Number of reads^a^	Transcripts	Average Length (bp)	N50 (bp)
Basal stem	69,206,815	32,769	552	653
Upper stem	73,841,682	33,705	596	739
Petiole	114,112,782	22,363	472	517
Leaf	64,524,479	30,474	555	667
Peduncle	90,677,154	37,183	576	703
CH flower	83,320,217	35,064	592	741
CL flower	102,746,962	35,795	615	769
Fruit	126,069,676	34,051	820	1255


*^a^Post-quality filtering, CH = chasmogamous, CL = cleistogamous.*

### Genome Annotation and Gene Ortholog Analysis

**FIGURE 2:**
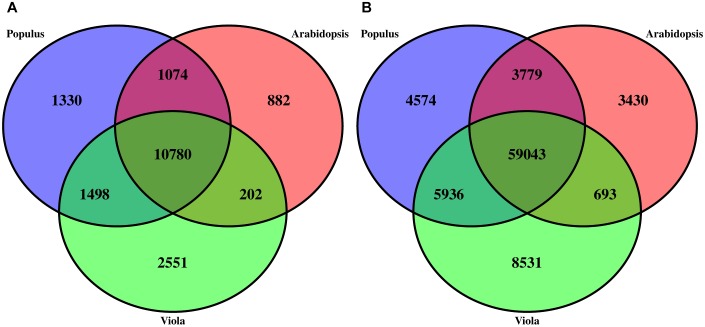
Three-way Venn diagrams **(A)** describing the distribution of shared and distinct protein clusters (orthogroups) from an OrthoMCL analysis of *V.pubescens* (green), *Arabidopsisthaliana* (red), and *Populustrichocarpa* (blue), and **(B)** the number of proteins within orthogroups.

Structural annotation of the *V. pubescens* genome was accomplished via the MAKER annotation pipeline, which generated 38,081 *ab initio* gene predictions. Functional annotation of the gene predictions were based on homology to Arabidopsis TAIR10, Swiss-Prot, or Pfam domain databases. Approximately 25% of the gene predictions were found to encode proteins with unknown function. Results of the structural annotation scoring are embedded within the publicly available GFF3 file^1^. To assess transcriptome integrity, the BUSCO Eudicotyledons_odb10 dataset was tested against the MAKER transcripts revealing 1,753 (82.6%) complete matches, 304 (14.3%) duplicated matches, 216 (10.2%) fragmented matches, and 152 (7.2%) missing matches. BUSCO results further justified the use of the *de novo* transcriptome assembly, as multiple genes were discovered that would otherwise not have been detected in a reference-based approach. Using the Markov Cluster algorithm implemented through OrthoMCL software (Li et al., 2003), orthologous clustering of *V. pubescens* proteins with the proteomes of *A. thaliana* and closely related model species, *Populus trichocarpa*, identified 18,317 orthogroups (groups of highly similar sequences), of which 2,551 were exclusive to *V. pubescens* (Figure 2A). *P. trichocarpa* was chosen for comparison with *V. pubescens* because of its membership in the closely related Salicaceae family. Within the orthogroups, 59,043 orthologs were identified among the three taxa (Figure 2B). As expected based on phylogenetic relationship, *V. pubescens* shared a larger number of orthologs with *P. trichocarapa* (5,936) than with *A. thaliana* (693).

### Comparison of Gene Expression Between Tissues

**FIGURE 3:**
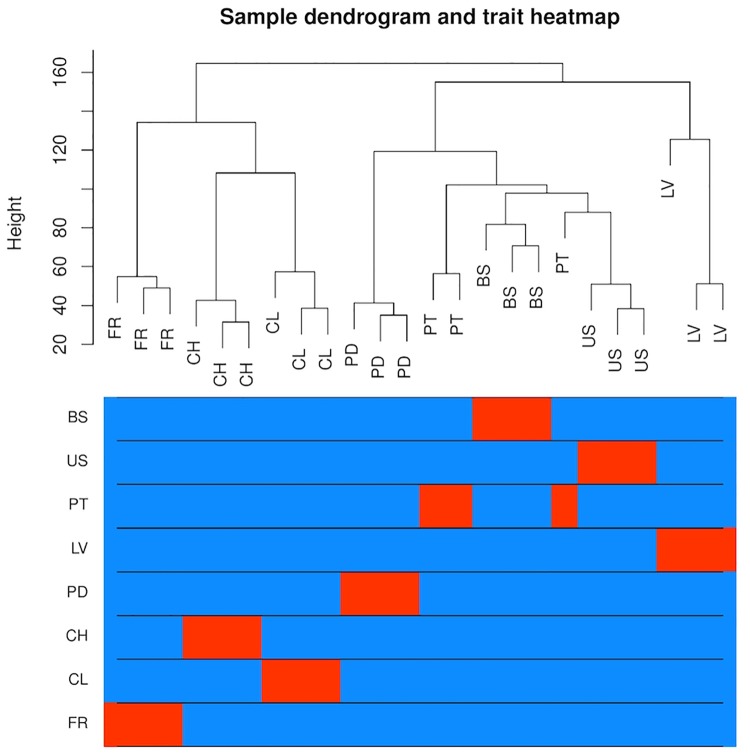
Analysis of gene expression across eight *V. pubescens* tissues through hierarchical clustering generated using Spearman’s correlation coefficients of log_2_CPM expression values from 7,815 predicted genes (CPM > 40 in ≥3 samples). The color intensity is proportional to the correlation of gene expression between tissue types with red denoting high correlation (BS = basal stem, US = upper stem, PT = petiole, LV = leaf, PD = peduncle, CH = chasmogamous flower, CL = cleistogamous flower, FR = fruit).

**FIGURE 4:**
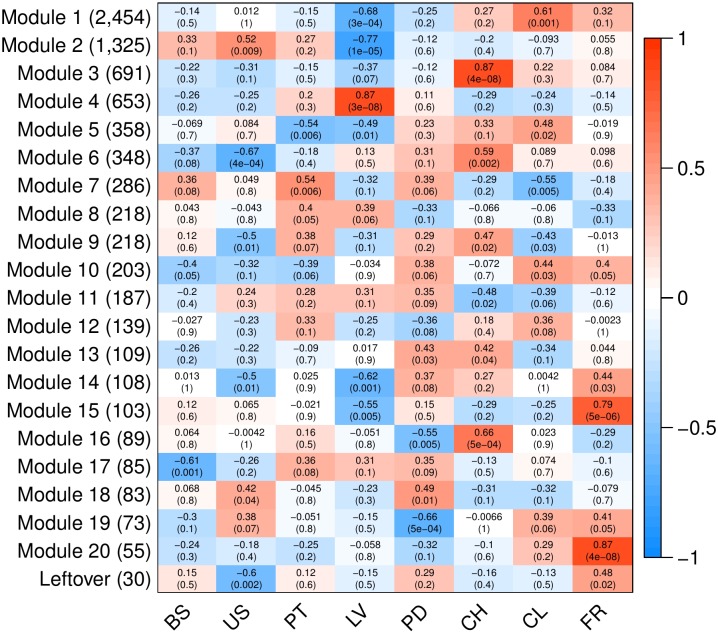
Heatmap of modules identified through WGCNA to contain highly correlated genes. Analyses were conducted using 7,815 predicted genes (CPM > 40 in ≥3 samples). Columns represent *V. pubescens* tissues (BS = basal stem, US = upper stem, PT = petiole, LV = leaf, PD = peduncle, CH = chasmogamous flower, CL = cleistogamous flower, FR = fruit) and their association with each module eigengene (rows). Values within cells correspond to the degree of correlation and significance (*p*-value in parentheses) between the expression of module eigengenes in the various tissues. The color scale indicates the correlation coefficient of each tissue and modules’ eigengene expression (blue = negative correlation, white = no correlation, red = positive correlation). On the y-axis, the number of genes included in each module is presented in parentheses.

To analyze variation in gene expression across tissue types, principal component analysis (PCA) (Supplementary Figure S2) and hierarchical clustering (Figure 3) were conducted using log_2_CPM (counts per million) values from genes with CPM greater than 40 in at least three replicates. A total of 7,815 of the predicted gene models met this cut-off. The reduced-dimension space via the first two principal components of the PCA show well-defined groups representative of each tissue type. Replicates of each tissue cluster in close proximity with the first-dimension separating tissues into photosynthetic and reproductive groups. Both clustering and separation on the first-dimension conform to biological expectations of variability and indicate that many genes are under tissue-specific regulation. Weighted Gene Co-Expression Network Analysis (WGCNA) was used to construct modules containing genes with highly correlated expression. The WGCNA sample dendrogram and trait heatmap support the findings of the PCA with similar clustering of tissue replicates (Figure 3). One petiole replicate is grouped closer to the basal stem and upper stem tissues, but it was not considered problematic given the similar biological function of petioles and stems. From the 7,815 genes used for WGCNA analysis, 20 gene co-expression modules were identified containing 7,785 genes. Modules represent genes with highly correlated expression and contained between 55 and 2,454 genes each. To visualize module expression patterns, eigengenes were calculated for each module and used to generate a heatmap of module-trait relationships (Figure 4). Genes that showed the highest correlation coefficient (≥0.89) with the module eigengene were considered hub genes, and of the 7,785 total genes, 146 were classified as such. Hub gene expression patterns for each module were also visualized through trend plots of normalized gene expression values (Supplementary Figure S3).

Like the PCA and trait heatmap, the co-expression within WGCNA modules indicates that many genes are expressed in a tissue-specific manner. For example, module 4 contains 653 genes, the majority of which are exclusively expressed in leaf tissue (Figure 4). The module is characterized by photosynthetic genes including hub genes with homology to *LOWPHOTOSYSTEM II ACCUMULATION 3* (*LPA3*), *CHLOROPLAST RNA BINDING PROTEIN* (*CRB*), *PHOTOSYSTEM II REACTION CENTER PsbP FAMILY PROTEIN*, *PHOTOSYSTEM II SUBUNIT Q-2* (*PSBQ-2*) and *FERREDOXIN-NADP(+)-OXIDOREDUCTASE 1* (*FNR1*). To provide additional support for module 4, the RNA-Seq data was used to generate a list of differentially expressed genes between leaves and all other tissue samples via EdgeR. A total of 2,003 genes were found to be significantly differentially expressed (FDR < 0.05, |log_2_FC|≥ 1). Approximately 74% of the genes in module 4 were differentially expressed in the leaves vs. all dataset, with 408 genes up-regulated in leaf tissue and 77 down-regulated. All of the hub genes in module 4 were captured in the differential expression data, with an average log_2_FC of 3.13. Gene Ontology (GO) enrichment analysis for the module 4 genes revealed significant fold enrichment (FE) of genes with GO terms for photosystem II repair (FE = 15.74), photosynthesis light harvesting (FE = 14.88), photosynthetic electron transport in photosystem I (FE = 14.69), photosynthesis dark reaction (FE = 14.39), photosynthetic electron transport chain (FE = 11.93) and photosynthesis light reactions (FE = 11.75) among others. The co-expression modules also highlight differences between similar tissues, such as modules 1, 3, 6 and 16, which contain genes that are largely expressed in either chasmogamous or cleistogamous flowers (Figure 4). These modules may hold insight into the genetic and developmental differences between chasmogamous and cleistogamous flowers in mixed breeding system species.

The chasmogamous/cleistogamous mixed breeding system is comprised of chasmogamous, open flowers that are showy and predominantly cross-pollinated, and cleistogamous, small, mechanically closed flowers that force self-pollination. Comparative studies in several species indicate that chasmogamous and cleistogamous flowers diverge early in their developmental pathways, with heterochrony as the suggested mechanism of divergence (Lord, 1979, 1982; Mayers and Lord, 1983, 1984; Minter and Lord, 1983). While the specific cues and underlying genetic basis of this mixed breeding system remain largely unknown, in most species, the timing and proportion of chasmogamous to cleistogamous flowers produced varies directly in response to environmental conditions (Lord, 1979; Corff, 1993; Cortes-Palomec and Ballard, 2006; Munguía-Rosas et al., 2013; Stojanova et al., 2014). Research in a number of mixed breeding system species has shown that chasmogamous flowers require greater investment of energy resources relative to cleistogamous flowers because of their larger size and petal and nectar production (Schemske, 1978; Waller, 1979). Because of this differential cost, chasmogamous flowering is generally more prevalent under favorable environmental conditions (Schemske, 1978; Waller, 1979). In most temperate herbs with the mixed breeding system, including *V. pubescens*, chasmogamous flowers are produced in spring before the canopy closes, and cleistogamous flowers are produced through summer and early fall following canopy closure and drastic reductions in light quantity and quality (Lord, 1981, 1984; Ballard et al., 2011). Various studies indicate that many mixed breeding system species shift their ratio of chasmogamous to cleistogamous flowers produced in response to these seasonal changes in light, with chasmogamy often increasing linearly with light intensity (Uphof, 1938; Cortes-Palomec and Ballard, 2006; Ballard et al., 2011). The temporal separation of chasmogamous and cleistogamous flowering in *V. pubescens*, with chasmogamy occurring close to the equinox and cleistogamy persisting through the summer solstice, may also reflect differences in photoperiod requirements for induction of each floral type.

This response to light signaling is highlighted by module 16, containing genes with increased expression in chasmogamous flowers (Figure 4). Many of these genes show homology to *A. thaliana* genes involved in floral development through regulation of circadian rhythmicity and photoperiodic signaling. Examples include *LATE ELONGATED HYPOCOTYL 1* (*LHY1*), *TIMING OF CAB EXPRESSION 1* (*TOC1*), *EARLY FLOWERING 3* (*ELF3*), and a hub gene homologous to *CONSTANS-LIKE 9* (*COL9*), which all act as transcriptional regulators of the circadian clock (Graf et al., 2017). In Arabidopsis, LHY1 and TOC1, along with LHY1 paralog CIRCADIAN CLOCK ASSOCIATED 1 (CCA1), constitute the core negative feedback loop of the circadian clock and regulate photoperiodic control of flowering by altering expression of multiple floral genes (Schaffer et al., 1998; Park et al., 2016; Oakenfull and Davis, 2017). ELF3 forms an additional feedback loop and physically interacts with photoreceptor PHYB to provide light input to the clock and has been proposed to indirectly activate *LHY1* and *CCA1* (Liu et al., 2001; Dixon et al., 2011). Many Arabidopsis clock mutants, including *lhy*, *cca1* and *elf3*, show altered photoperiodic responses and cause early flowering phenotypes even under non-inductive conditions (Hicks et al., 2001; Park et al., 2016). One major mediator between the circadian clock and floral genes is the transcription factor CONSTANS (CO), a promoter of flowering through activation of *FLOWERING LOCUS T* (*FT*), a known facilitator of vegetative to floral transition through the photoperiodic pathway (Searle and Coupland, 2004; Jung et al., 2007). The protein product of hub gene *COL9* represses *CO* and subsequently *FT* expression, which delays expression of *LEAFY* (*LFY*) and *APETELA 1* (*AP1*) floral integrators and prevents precocious flowering (Castillejo and Pelaz, 2008; Wickland and Hanzawa, 2015). This may represent a significant difference between the development of chasmogamous and cleistogamous flowers, as unlike chasmogamous flowers, cleistogamous flowers are marked by precocious development and sexual maturation (Lord, 1979, 1982; Mayers and Lord, 1983, 1984). GO enrichment for module 16 genes identified significant enrichment of circadian rhythm (FE = 12.04) and rhythmic process (FE = 11.22).

Module 3 also contains genes with predominant expression in chasmogamous flowers (Figure 4). Many of these genes have roles in regulating floral development through circadian and photoperiodic signaling, with hub genes homologous to *TOPLESS-RELATED 3* (*TPR3*) and *RELATED TO AP2-4* and -*6L* (*RAP2.4*, *RAP2.6L*). TPR3 acts as a co-repressor in multiple pathways including circadian clock entrainment and flowering time regulation (Liu and Karmarkar, 2008; Causier et al., 2012; Wang et al., 2013). Recently, TPR3 was found to interact with TOE1 and TOE2 to transcriptionally repress *FT* and, similar to the repressing activity of COL9 in module 16, leads to delayed flowering (Jung et al., 2007; Castillejo and Pelaz, 2008; Causier et al., 2012). Members of the Arabidopsis ERF/AP2 transcription factor family, including *RAP2.4*, have also been suggested to play a regulatory role in flowering through light signaling. Accumulation of *RAP2.4* mRNA is significantly reduced under all wavelengths, and overexpression of *RAP2.4* promotes early flowering (Lin et al., 2008). GO enrichment for module 3 identified several homologs involved in floral organ morphogenesis (FE = 4.86). Of specific interest in this enrichment group were homologs of *APETALA 3* (*AP3*), *PISTILLATA* (*PI*), and *BIG PETAL* (*BPEp*). The ABC model describes how three classes of homeotic genes direct floral organ formation with A-class genes *APETALA 2* (*AP2*) and *APETALA 1* (*AP1*) overlapping with B-class genes *APETALA 3* (*AP3*) and *PISTILLATA* (*PI*) to dictate petal development (Weigel and Meyerowitz, 1994; Wollmann et al., 2010). The presence of these genes is consistent with module 3 containing genes primarily expressed in chasmogamous tissue, as unlike cleistogamous flowers, chasmogamous flowers contain petals. This is also true for *BPEp*, a transcription factor that is preferentially expressed in petals and involved in regulating petal growth (Varaud et al., 2011). For module 3, additional GO enrichment was observed for terms pertaining to single-organism carbohydrate catabolic process (FE = 5.27), carbohydrate catabolic process (FE = 3.09), single-organism carbohydrate metabolic process (FE = 2.36), carbohydrate derivative metabolic process (FE = 2.09), and carbohydrate metabolic process (FE = 1.85). Carbohydrate metabolism is regulated by light and the circadian clock (Graf et al., 2010; Kim et al., 2017), and the enrichment of terms pertaining to carbohydrate metabolism may relate directly back to the differences in light and resource requirements between chasmogamous and cleistogamous flowers. This relationship may also be represented in module 6, with upregulated chasmogamous expression (Figure 4) and significant GO enrichments for cellular response to phosphate starvation (FE = 6.59), response to starvation (FE = 5.14), response to nutrient levels (FE = 4.62), response to high light intensity (FE = 4.94) and cellular response to light stimulus (FE = 4.5). From the RNA-Seq, a differential expression data set comparing chasmogamous and cleistogamous tissues was created, and a total of 2,898 genes were found. Of these genes, 1,363 were significantly differentially expressed between chasmogamous and cleistogamous flowers (FDR < 0.05, |log_2_FC|≥ 1). These genes were filtered to include only those specific to modules 3, 6, and 16 leading to 465 differentially expressed genes of which 441 were upregulated in chasmogamous flowers.

In contrast to modules 3, 6 and 16, the expression of module 1 genes is largely increased in cleistogamous flowers (Figure 4). Module 1 contains 2,454 genes of which 401 were found to be significantly differentially expressed between chasmogamous and cleistogamous tissues (FDR < 0.05, |log_2_FC|≥ 1). Of the 401 differentially expressed genes, 322 were upregulated in cleistogamous flowers. Included in those differentially expressed genes were hub genes with homology to *CHROMATIN REMODELING 1* (*CHR1*) and *INCURVATA 2* (*ICU2)*. CHR1, a chromatin-remodeling ATPase, exemplifies the differential regulation of genes and processes controlling DNA topology and, more broadly, the regulation of gene expression at the epigenetic level (Lyons and Zilberman, 2017). In Arabidopsis, ICU2 has also been suggested to play a role in epigenetic inheritance and chromatin packaging and regulates multiple genes involved in flowering time and floral meristem and organ identity (Barrero et al., 2007). The possibility of epigenetic regulation is supported by the presence of GO enrichments for heterochromatin organization (FE = 6.13), DNA methylation on cytosine (FE = 5.84), DNA packaging (FE = 3.73), chromatin assembly (FE = 3.67), negative regulation of gene expression – epigenetic (FE = 3.15), DNA conformation change (FE = 2.98), DNA methylation (FE = 2.73) and regulation of gene expression – epigenetic (FE = 2.70). Changes in the epigenetic regulation of gene expression may represent a crucial step in the induction of cleistogamous flowering as previous results have indicated that floral transition is largely controlled by chromatin-mediated gene silencing (He and Amasino, 2005; Reyes, 2006; Barrero et al., 2007).

Module 1 also contains a differentially expressed gene with homology to *ARGONAUTE 5* (*AGO5*). The Argonaute proteins are involved in RNA silencing, and in Arabidopsis, *AGO5* is solely expressed in reproductive tissues (Kapoor et al., 2008). The upregulation of *AGO5*’s homolog in cleistogamous tissues may indicate a significant alteration in the regulatory landscape between the two flower types. This alteration is also emphasized by additional module 1 GO enrichments gene silencing by miRNA (FE = 3.37), regulation of gene silencing (FE = 2.96), posttranscriptional gene silencing (FE = 2.69), production of small RNA involved in gene silencing by RNA (FE = 2.62), posttranscriptional gene silencing by RNA (FE = 2.56), gene silencing (FE = 2.51) and gene silencing by RNA (FE = 2.31). Of note, an *APETALA 2* (*AP2*) homolog, with decreased expression in cleistogamous tissues, was found in both module 1 and the differential expression data. AP2, a member of the AP2-like transcription factors, represses flowering by inhibiting expression of *FT* and various floral meristem identity genes. *AP2* is co-repressed by MICRORNA 172 (miR172), a main component of the “heterochronic pathway” in flowering plants (Aukerman and Sakai, 2003; Geuten and Coenen, 2013; Hong and Jackson, 2015). Recently, the *Cleistogamy1* (*Cly1*) gene in barley was identified as a homolog of *AP2* and encodes a transcription factor with two AP2 domains and a putative miR172 binding site (Nair et al., 2010). Single nucleotide substitution at the miRNA172 target site leads to cleistogamous flowering in barley, suggesting that genes containing miR172 sites, like *AP2*, are regulated by miR172-directed mRNA cleavage and/or translational repression (Aukerman and Sakai, 2003; Chen, 2004; Nair et al., 2010). The down-regulation of an *AP2* homolog in cleistogamous tissues of *V. pubescens* may signify heterochronic differences between chasmogamous and cleistogamous flowers. Additionally, *AP2*’s homology to *Cly1* and regulation via miRNA processes signify an apparent change in gene silencing in cleistogamous flowers that is generally characteristic of module 1. Another possible explanation for the down regulation of an *AP2* homolog in cleistogamous tissues is *AP2*’s role in conferring petals. Like *ap2* mutants, cleistogamous flowers lack petals.

### *V. pubescens* Cyclotide Diversity

To identify putative cyclotides in the *V. pubescens* genome, nucleotide and protein sequences for 134 published cyclotides were extracted from Burman et al. (2015) and NCBI and CyBase repositories. The cyclotide sequences were derived from eight Violaceae genera, including 27 diverse violet species, as well as three distant Rubiaceae genera/species for outgroups (Supplementary Table S3). The sequences were queried against *V. pubescens* BLAST nucleotide and protein databases resulting in >3500 hits. The hits were filtered to remove redundancy between queries leading to 81 putative *V. pubescens* cyclotides. Assuming that 81 is the average number of cyclotides in a *Viola* species, with 580–620 *Viola* genera alone and ∼1,100 Violaceae species worldwide, it is possible that Violaceae contains more than 89,000 cyclotides. This is supported by Hellinger et al. (2015), who had estimated that the number of individual Violaceae cyclotides to be as many as 150,000 based on *Viola tricolor* transcriptome mining and mass spectrometry. To validate the *V. pubescens* cyclotides, their 81 protein sequences, along with the 134 protein sequences from other species (Supplementary Data Sheet S1), were aligned using MUSCLE. The MUSCLE output was then re-aligned in trimAl to remove poorly aligned regions. The most conserved region (GIP-CGES-CV-WIP-C) was queried in the Pfam database resulting in a significant domain match to the cyclotide family (e-value = 3.8e-07, bit score = 30). The trimAl alignment was then used to generate a maximum-likelihood protein cladogram through PhyML (Supplementary Figure S4). The dispersed nature of the *V. pubescens* cyclotides throughout the cladogram and intermingling among other Violaceae and Rubiaceae species provides evidence that the *V. pubescens* genome contains many diverse cyclotides. The branch divisions may be reflective of cyclotide subfamily groupings, but the sequencing coverage of cyclotides used in the analysis was insufficient to accurately discern subfamilies. While the protein cladogram shows terminal groupings with substantial bootstrap values, there is no appreciable basal support. This lack of support may be because entirely different proteins, not orthologs, were used in the analysis, and the majority of the queried sequences were translated from partial genes. To evaluate the expression of the 81 *V. pubescens* cyclotide transcripts, read counts per gene were calculated with quantMode (htseq-count option –s reverse) in STAR. Counts were used to generate a read counts per gene matrix and values were transformed to logCPM. Expression patterns were diverse between both *V. pubescens* cyclotide transcripts and tissue types (Supplementary Data Sheet S2).

## Conclusion

In the present study, we describe the *de novo* assembly and annotation of the *V. pubescens* genome from 26.6 Gbp of short-read DNA-Seq. Gene structural annotation was aided by the use of RNA-Seq transcript assemblies that were derived from a diverse set of eight *V. pubescens* tissues as well as protein sequences from *A. thaliana* (TAIR10) and Swiss-Prot excluding *Viola* sequences. Despite the fact that the short read genome assembly contained 161,038 contigs/scaffolds, 38,081 gene models were identified in the genome assembly. We have highlighted tissue-specific gene expression through PCA and hierarchical clustering, and WGCNA analyses revealed 20 co-expression modules. The gene co-expression within modules indicates genes are expressed in a tissue-specific manner, and the functional annotations of these genes conform to biological expectations, demonstrating relevance of the expression data and supporting the genome’s functional annotations. Modules with increased expression in chasmogamous flowers contained many genes involved in the control of floral transition. These genes may reflect a critical regulation of chasmogamous flowering to occur when conditions will maximize reproductive success. Because cleistogamous flowers are obligate self-fertilizers and less energetically costly to produce, their floral transition may not be as tightly regulated. The presence of circadian clock and photoperiodic flowering genes in predominantly chasmogamous expressed modules, especially genes that repress precocious flowering, may also emphasize heterochronic differences in chasmogamous and cleistogamous flowering in response to distinct light and resource requirements. Additional heterochronic differences appear in the increased expression of genes involved in chromatin remodeling and gene silencing in cleistogamous tissues. This gene silencing may hold insight into the role of *AP2* and miR172 in conferring cleistogamy, specifically in non-grass species, a largely unexplored research area. The *V. pubescens* genome also facilitated *in silico* identification of 81 novel and diverse cyclotides. The expression of these 81 cyclotides reveals unique expression patterns both between cyclotides and tissues. This is the first genome-wide identification of cyclotides within a Violaceae species, and it suggests that within all of Violaceae there may exist more than 89,000 cyclotides. In summary, the *V. pubescens* draft genome represents the first *Viola* genome and provides valuable genomic and transcriptomic resources for future molecular genetic studies. This includes studies investigating mixed breeding, cyclotide presence and expression, and other life history traits not characterized by current model systems.

## Materials and Methods

### DNA Preparation and Sequencing

The *V. pubescens* genome size was estimated by flow cytometry. Fresh leaf samples of *V. pubescens* and reference samples of *P. trichocarpa* were shipped to the Flow Cytometry and Imaging Core laboratory at Virginia Mason Research Center. Four DNA samples were extracted and analyzed per species with chicken erythrocyte nuclei used as the internal reference sample. To sequence the *V. pubescens* genome, genomic DNA was extracted from leaf tissue of native *V. pubescens* plants located at Sells Park, a mixed mesophytic forest in Athens County, Ohio, 45701 (39°20′40.6′′N 82°04 ′31.9′′W). Extractions were accomplished using a DNeasy Plant Mini Kit (Qiagen, Inc.). DNA quality and concentration were assessed using a Bioanalyzer (Agilent, Inc.), and four libraries were sequenced at The Ohio State University Nucleic Acid Shared Resource facility on Illumina HiSeq 2000 (Illumina, Inc.) with paired-end, 100 bp chemistry. For further genome size estimation, the raw DNA reads were trimmed using Sickle (v.1.33)^2^ and only reads of quality 25 or higher were considered for analysis. A total of 260,945,571 read pairs remained post-trimming. The k-mer distribution of these reads was determined using Jellyfish (v.2.2.8) (Marçais and Kingsford, 2011) and k-mer sizes of 17, 31, 49, 63, and 79. Genome heterozygosity, length, repeat length, and unique length were then analyzed through Genomescope (v.1.0) (Vurture et al., 2017). A k-mer size of 63 provided the optimal fit to the Genomescope model and was used for final genome size estimation.

### Genome Assembly and Annotation

FastQC^3^ (v0.11.2) was employed to assess read quality both before and after trimming adapters with Cutadapt (v.1.8.1) (Martin, 2011). Following adapter trimming, two of the four libraries failed per base sequence quality, per sequence quality scores, and per base N content quality metrics. Sequences < 500 bp and/or containing low quality bases were removed from all libraries, and the filtered reads were assembled using the ABySS (v1.5.2) de Bruijn graph assembler (Simpson et al., 2009). For assembly, k-mer sizes of 69, 79, and 89 were tested for assembly of all four libraries as well as just the two libraries that passed FASTQC quality control. Based on the ABySS output (Supplementary Table S4), the assembly from the two superior libraries and a k-mer of 79 was selected. To identify contaminant reads within the assembly, reads were mapped back to contigs to determine average coverage via Blobology (Kumar et al., 2013), and a GC content vs. coverage plot was visualized using Blobtools (Laetsch and Blaxter, 2017). To analyze genome completeness and contiguity of the ABySS assembly, homology to the CEGMA (Parra et al., 2007) and BUSCO (Simão et al., 2015) datasets was tested. CEGMA screened the *V. pubescens* assembly against a collection of 248 core eukaryotic genes (CEGs), and BUSCO compared the genome assembly against a collection of 2121 single-copy plant orthologs from the OrthoDB (Eudicotyledons_obd10) database. The *de novo* genome assembly was masked using RepeatMasker (v4.0.5) and default parameters. Structural annotation was accomplished using the MAKER structural annotation pipeline (Campbell et al., 2014). RNA-Seq assemblies and protein evidence from TAIR10 (Berardini et al., 2015) and Swiss-Prot (Bairoch and Apweiler, 2000) were aligned to the genome and the aligned regions were used as input to train SNAP (Leskovec and Sosič, 2016) and AUGUSTUS (Stanke and Morgenstern, 2005) *ab initio* gene prediction programs. Outputs from both programs were synthesized into final gene annotations with evidence-based quality values allowing for downstream annotation management. Functional descriptions of the MAKER standard gene predictions were based on homology to either TAIR10, Swiss-Prot, or Pfam domain. Gene predictions with putative transposon related function were removed from the final genome annotation.

### RNA Preparation and Sequencing

RNA was extracted from eight tissues of native *V. pubescens* populations^4^ located in Sells Park, Athens County, Ohio, 45701 (39°20′40.6′′N 82°04′31.9′′W). Tissues included basal stem, petioles, leaf blades, upper stem, peduncle, immature buds, immature fruit and mature chasmogamous and cleistogamous flowers. Plant material was harvested in the field (Sells Park, Athens County, OH, 45701; 39°20′40.6′′N 82°04′31.9′′W) and flash frozen in liquid nitrogen. Approximately 100 mg of RNA was extracted per sample using an RNeasy Plant Mini Kit (Qiagen, Inc.) as per manufacturer’s instructions. Three replicates of each tissue type were extracted with three plants used per biological replicate, resulting in a total of 24 samples. RNA integrity and concentration were evaluated on a Bioanalyzer (Agilent, Inc.). Samples with RNA integrity scores ≥ 8 were sent to the Genomics Core Facility at Michigan State University (East Lansing, MI) for library preparation and sequencing via Illumina HiSeq 2500 (Illumina, Inc.) with single-end, 50 bp chemistry.

### Transcriptome Assembly and Gene Co-expression

RNA-Seq reads were cleaned using Trimmomatic (v.0.32) (Bolger et al., 2014), and low-quality sequences and adaptors were removed. RNA-Seq data sets from replicated samples were assembled using Trinity (v.2.0.6) (Grabherr et al., 2011) with default parameters. Transcripts were mapped to the assembled *V. pubescens* genome and read counts per gene were calculated with quantMode (htseq-count option –s reverse) in STAR (v.2.4.2a) (Dobin et al., 2013). For WGCNA analysis, a transcript was considered expressed if the CPM was greater than 40 in at least three of the 24 RNA-Seq datasets. To identify modules of highly correlated genes, the WGCNA R package was used (Langfelder and Horvath, 2008) with a soft threshold value β of 10 and a treecut value of 0.30. All other parameters used default settings. Eigengenes representing the overall expression patterns within each module were calculated. To visualize these expression patterns, eigengenes were used to generate a heatmap with correlation and significance values for each module and tissue. Within modules, genes with the highest correlation coefficient (≥0.89) with the module eigengene were considered hub genes, and the z-scores of expression data for individual module’s hub genes were plotted using the ggplot2 package of R (Wickham, 2009).

### Differential Expression and Gene Ontology Enrichment of Module Genes

Reads per gene were re-implemented from the STAR output used in gene co-expression analyses. Genes with CPM > 40 in at least three replicates of each comparison were considered for differential expression analysis. Differential expression was determined using the generalized linear model likelihood test within the Empirical Analysis of Digital Gene Expression Data in R package EdgeR (v.3.16.5) (Robinson et al., 2010). False discovery rate (FDR) (≤0.05) and log-fold change (|log_2_FC|≥ 1) were used to determine significantly expressed genes. GO (Ashburner et al., 2000) enrichments were performed using the Python based tool, Orange (v.3.7.0) (Demšar et al., 2013). For the reference dataset, only *V. pubescens* genes with annotation matches to *A. thaliana* orthologs were included. GO enrichments were filtered to include GO terms with an FDR ≤ 0.05 and *p*-value ≤ 0.05 via binomial significance. To exclude poorly annotated and/or broad GO terms, at least 5 reference genes had to be present within each GO term.

### Cyclotide Diversity

Protein and nucleotide sequences of 134 published cyclotides were extracted from NCBI (NCBI Resource Coordinators, 2012) and CyBase (Wang et al., 2008). The species from which the sequences were identified are provided in Supplementary Table S3, and their queried protein sequences are available in Supplementary Data Sheet S1. Both nucleotide and protein sequences for the 134 published cyclotides were queried against the *V. pubescens* genome through BLAST (v.2.6.0) (Altschul et al., 1990). Transcriptomes of *V. tricolor* and *Viola canadensis* were downloaded from the 1KP Consortium (Matasci et al., 2014) and used to generate BLAST databases. The nucleotide sequences for the 81 *V. pubescens* cyclotides were queried against the *V. tricolor* and *V. canadensis* databases yielding redundant hits to those previously mined from NCBI and CyBase. The protein sequences of the 134 queried cyclotides and the 81 identified in *V. pubescens* were aligned through MUSCLE (v.3.8.31) (Edgar, 2004) and trimAL (v.1.4.1) (Capella-Gutiérrez et al., 2009). For the maximum likelihood protein cladogram, the trimAL alignment was used as input for ProtTest (v.3.4.2) (Darriba et al., 2011) to select a model of amino acid replacement. Branch lengths and topologies were calculated with PhyML (v.3.1) (Guindon et al., 2010) using the VT amino acid substitution model, estimated portions of invariable sites, estimated Γ-distribution shape parameter, eight substitution rate categories, estimated amino acid frequencies, 100 bootstrap replicates, and the best of nearest neighbor interchange (NNI) and subtree pruning and regrafting (SPR). The cladogram was visualized and color-coded in Geneious with different colors representing each genera (color key is listed in Supplementary Table S3).

## Data Availability

The *Viola pubescens* var. scabriuscula Whole Genome Shotgun Sequencing project has been deposited at DDBJ/ENA/GenBank under the accession NBIL00000000 (version NBIL00000000.1) (Sternberger et al., 2015).

## Author Contributions

AS made substantial contributions to the project concept/design and acquisition of data, performed all field and wet lab work besides DNA and RNA sequencing, conducted the differential expression, GO, OrthoMCL, and cyclotide protein cladogram analyses, and also interpreted manuscript data at large and drafted the majority of the manuscript. MB performed the ABySS *de novo* genome assembly, structural and functional annotation of the genome assembly, wrote scripts for the WGCNA analysis, generated the initial OrthoMCL data set, and also revised the final manuscript version. CK assisted with tissue harvesting, analyzing the RNA-Seq data, interpretation of the WGCNA analysis, writing scripts for differential expression analysis, and manuscript drafting. KC was responsible for each of the transcriptome assemblies and participated in data interpretation and manuscript drafting/revisions. HB located *V. pubescens* populations for tissue harvesting and helped specifically with interpreting the cyclotide cladogram. HB and SW directed the project, acquired funding, and made substantial contributions to data interpretation, manuscript drafting, and manuscript revisions.

## Conflict of Interest Statement

The authors declare that the research was conducted in the absence of any commercial or financial relationships that could be construed as a potential conflict of interest.

## Footnotes

1 https://doi.org/10.6084/m9.figshare.7409360.v1

2 Joshi, N. A., and Fass, J. N. Sickle: A sliding-window, adaptive, quality-based trimming tool for FastQ files (version 1.33). Available at: https://github.com/najoshi/sickle/.

3 Babraham Bioinformatics – FastQC a quality control tool for high throughput sequence data. Available at: http://www.bioinformatics.babraham.ac.uk/projects/fastqc/.

4 Representative seeds have been deposited at the Ornamental Plant Germplasm Center (OPGC) and are available upon reasonable request (https://opgc.osu.edu/how-order-germplasm).
