# Corrigendum: Transcriptomics Identifies Modules of Differentially Expressed Genes and Novel Cyclotides in *Viola pubescens*

**DOI:** 10.3389/fpls.2019.00278

**Published:** 2019-03-18

**Authors:** Anne L. Sternberger, Megan J. Bowman, Colin P. S. Kruse, Kevin L. Childs, Harvey E. Ballard, Sarah E. Wyatt

**Affiliations:** ^1^Department of Environmental and Plant Biology, Ohio University, Athens, OH, United States; ^2^Department of Plant Biology, Michigan State University, East Lansing, MI, United States; ^3^Interdisciplinary Molecular and Cellular Biology Program, Ohio University, Athens, OH, United States

**Keywords:** *Viola pubescens*, transcriptomics, gene co-expression analysis, cyclotides, genome assembly, mixed breeding, chasmogamous, cleistogamous

In our original article, there was a mistake in the legend for **Figure 1** as published. The descriptions of the figure labels (A) and (B) were switched. The correct legend appears below.

“*Viola pubescens* var. scabriuscula bearing **(A)** cleistogamous and **(B)** chasmogamous flowers. Photographs were taken over native populations located in Sells Park, Athens County, Ohio, 45701 (39°20′40.6″N 82°04′31.9″W).”

Additionally, there was a mistake in Table 1 as published. In the first column, “# of scaffolds > 10 kbp” should be “# of scaffolds > 1 K nt.” The corrected Table 1 appears below.

**Table 1:** Summary statistics of the *V. pubescens* genome assembly via ABySS and CEGMA and BUSCO assessments of genome completeness.

# of scaffolds	157,722
Total size of scaffolds (bp)	318,370,682
Longest scaffold (bp)	86,685
# of scaffolds > 1 K nt	80,885
N50 length (bp)	3,500
Average length of break (>25 N's) between contigs in scaffold	45
Scaffold %N	0.06
Percent in scaffolded contigs	3.2
Percent in unscaffolded contigs	96.8
CEGMA Partial (%)	94, *n* = 248
CEGMA Complete (%)	76, *n* = 248
BUSCO	C: 79.7% [D:13.5%], F: 8.9%, M: 11.4%, *n* = 2121

*C, complete; D, duplicated; F, fragmented; M, missing; n, gene number*.

The authors apologize for these errors and state that they do not change the scientific conclusions of the article in any way. The original article has been updated.

