# Supplementary material for: Transcriptomics Identifies Modules of Differentially Expressed Genes and Novel Cyclotides in Viola pubescens
Source: Front Plant Sci. 2019 Feb 15;10:156. doi: 10.3389/fpls.2019.00156 (PMC6384259; doi:10.3389/fpls.2019.00156)
Supplement: Supplementary file 3 [file Data_Sheet_3.docx]

Supplementary Material

**Transcriptomics Identifies Modules of Differentially Expressed Genes and Novel Cyclotides in *Viola pubescens***

**Anne L. Sternberger, Megan J. Bowman, Colin P.S. Kruse, Kevin L. Childs, Harvey E. Ballard, and Sarah E. Wyatt^*^**

*** Correspondence:** Dr. Sarah Wyatt: [wyatts@ohio.edu](mailto:wyatts@ohio.edu)

# Supplementary Figures and Tables

## Supplementary Figures

### Supplementary Figure 1


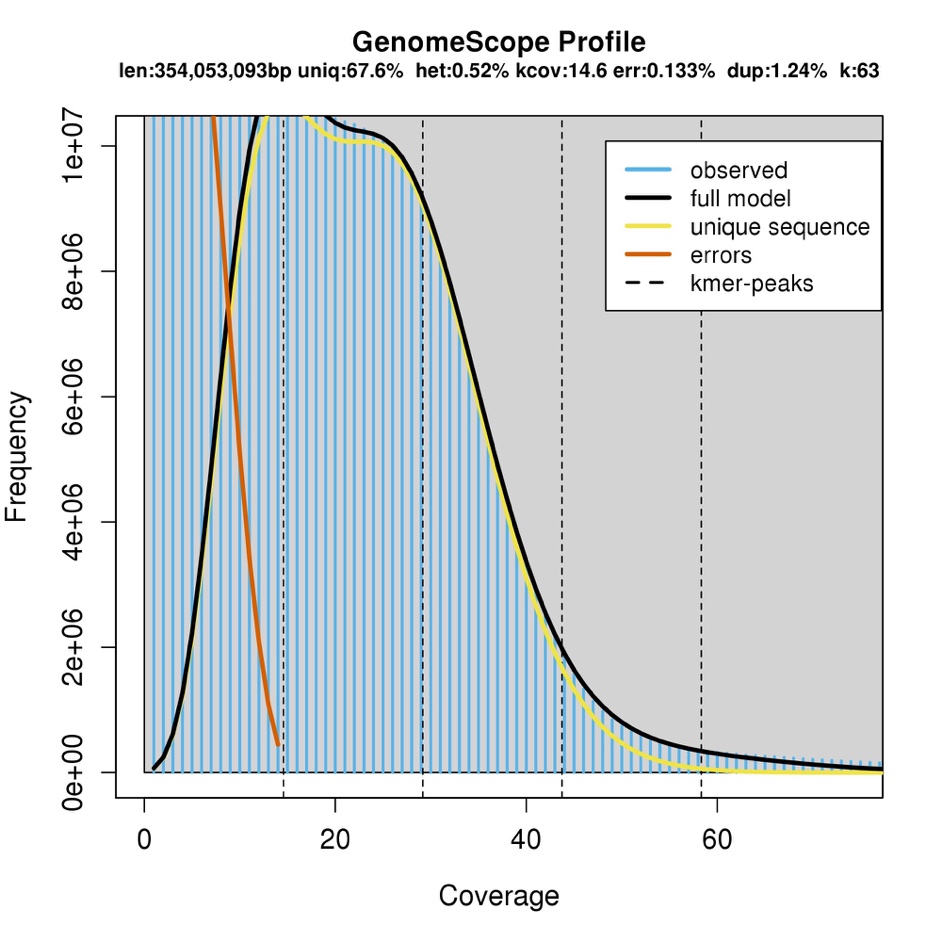


Figure S1. Estimation of total genome length (len), unique reads (uniq), heterozygosity (het), coverage for heterozygous bases (kcov), read error rate (err), and average rate of read duplications (dup) through GenomeScope (Vurture et al., 2017) using a Jellyfish (Marçais and Kingsford, 2011) k-mer distribution approach and a k-mer size (k) of 63.

### Supplementary Figure 2


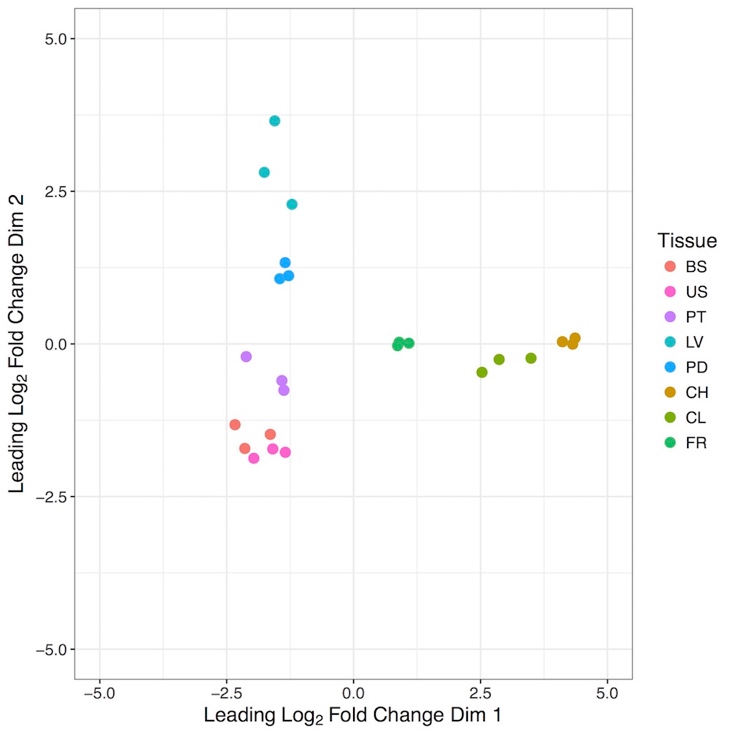


Figure S2. Analysis of variation in gene expression across eight *Viola pubescens* tissues through principal component analysis (PCA) of log_2_CPM values for each tissue replicate. Expression values from 7,815 predicted genes were included in the PCA. BS=basal stem, US=upper stem, PT=petiole, LV=leaf , PD=peduncle, CH=chasmogamous flower, CL=cleistogamous flower, FR=fruit.

### Supplementary Figure 3


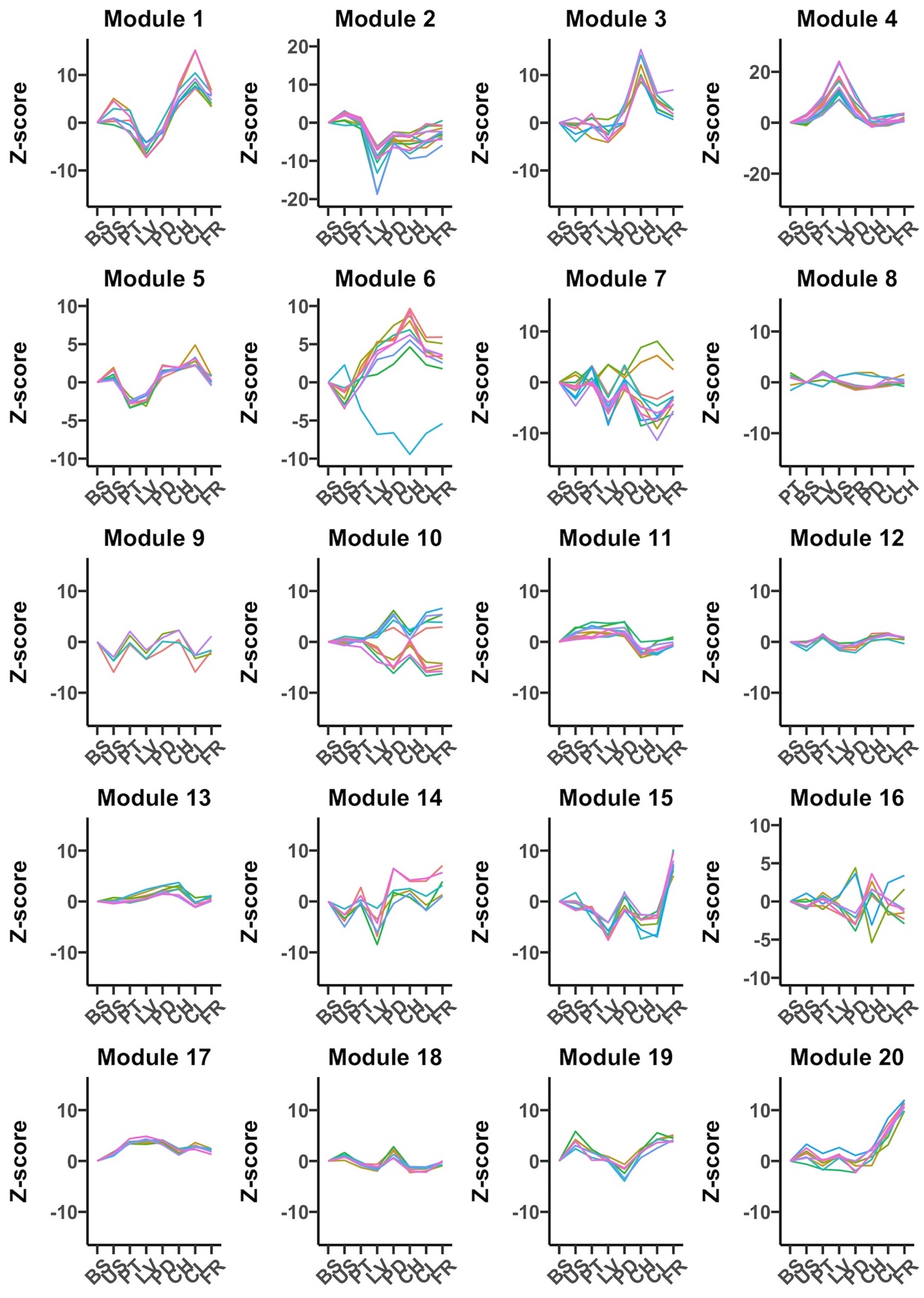


Figure S3. Trend plots of normalized gene expression values (z-scores) for the hub genes in each of 20 co-expression modules identified through weighted gene co-expression analyses on 7,815 *V. pubescens genes* (CPM > 40 in ≥ 3 samples). BS = basal stem, US = upper stem, PT = petiole, LV = leaf, CH = chasmogamous, CL = cleistogamous, FR = fruit.

### Supplementary Figure 4


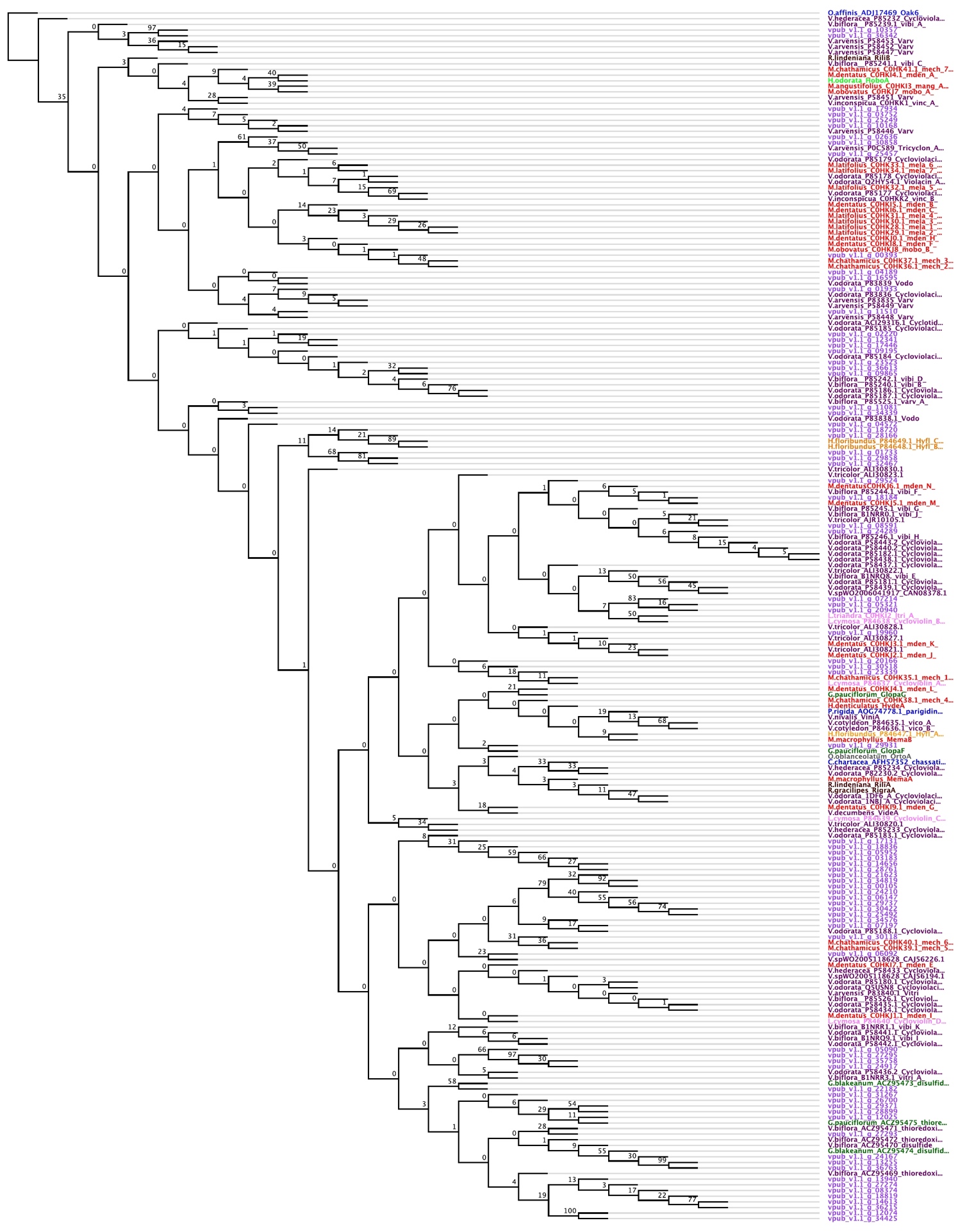


Figure S4: Maximum-likelihood protein cladogram containing 215 cyclotide sequences from eight Violaceae genera, including 27 diverse violet species, as well as three Rubiaceae genera/species used as cladogram outgroups. The cladogram was generated through PhyML using the VT amino acid substitution model, estimated portions of invariable sites, estimated Γ-distribution shape parameter, eight substitution rate categories, estimated amino acid frequencies, 100 bootstrap replicates, and the best of nearest neighbor interchange (NNI) and subtree pruning and regrafting (SPR). The cladogram was visualized and color-coded in Geneious with different colors representing each genera (color key listed in Table S1). Branches represent bootstrap support and are not proportional to amino acid changes.

## Supplementary Tables

### Table S1

Table S1. Species names and corresponding NCBI accession numbers of nine, publicly available *Viola* transcriptomes.

| Species | NCBI Accession |
| --- | --- |
| *Viola acuminata* | GFWD00000000 |
| *Viola albida* | GFWC00000000 |
| *Viola baoshanensis* | PRJNA268667 |
| *Viola canadensis* | ERX2099460 |
| *Viola mandshurica* | GFWG00000000 |
| *Viola orientalis* | GFXR00000000 |
| *Viola tricolor* | ERX2099459 |
| *Viola uliginosa* | PRJNA257024 |
| *Viola verecunda* | GFWF00000000 |

### Table S2

Table S2. Estimations of *V. pubescens* genome heterozygosity, haploid length, repeat length, unique reads length, model fit, and read error rate generated through GenomeScope (Vurture et al., 2017) using a k-mer distribution approach via Jellyfish (Marçais and Kingsford, 2011). K-mer sizes 17, 31, 49, 63, and 79 were tested and 63 was selected based on model fit.

|  | k17 | k31 | k49 | k63 | k79 |
| --- | --- | --- | --- | --- | --- |
| Heterozygosity (%) | 0.69 | 0.63 | 0.54 | 0.52 | 0.64 |
| Genome haploid length (bp) | 326,879,345 | 350,122,653 | 350,914,765 | 353,790,935 | 361,718,559 |
| Genome repeat length (bp) | 192,410,454 | 151,066,652 | 121,846,052 | 114,746,721 | 120,826,214 |
| Genome unique length (bp) | 134,468,891 | 199,056,001 | 229,068,713 | 239,044,214 | 240,892,345 |
| Model fit (%) | 86.09 | 90.73 | 93.89 | 95.70 | 92.96 |
| Read error rate (%) | 0.14 | 0.15 | 0.14 | 0.13 | 0.10 |

### Table S3

Table S3. Species with published cyclotide sequences used as queries for *in silico* identification of cyclotide sequences in the *V. pubescens* genome and phylogenetic analysis through PhyML.

| Genus | Species | GenBank Accessions or Publication | Color in Fig. S3 |
| --- | --- | --- | --- |
| **Chassalia* | *chartacea* | AFH57352 | Blue |
| *Gloeospermum* | *blakeanum* | ACZ95473, ACZ95474 | Green |
| *Gloeospermum* | *pauciflorum* | Glopa F and Glopa G in Burman et al. (2015), ACZ95475 | Green |
| *Hybanthus* | *denticulatus* | Hyde A in Burman et al., 2015 | Orange |
| *Hybanthus* | *floribundus* | P84647, P84648, P84649 | Orange |
| *Hymenanthera* | *oborata* | Hobo A in Burman et al. (2015) | Light green |
| *Leonia* | *cymosa* | P84637, P84638, P84639, P84640 | Pink |
| *Leonia* | *triandra* | C0HKI2 | Pink |
| *Melicytus* | *angustifolius* | C0HKI3 | Red |
| *Melicytus* | *chathamicus* | C0HK35, C0HK36, C0HK37, C0HK38, C0HK39, C0HK40, C0HK41 | Red |
| *Melicytus* | *dentatus* | C0HKJ0, C0HKJ1, C0HKJ2, C0HKJ3, C0HKJ4, C0HKJ5, C0HKJ6, C0HKI4, C0HKI5, C0HKI6, C0HKI7, C0HKI8, C0HKI9 | Red |
| *Melicytus* | *latifolius* | C0HK28, C0HK29, C0HK30, C0HK31, C0HK32, C0HK33, C0HK34 | Red |
| *Melicytus* | *macrophyllus* | Mema A and Mema B in Burman et al. (2015) | Red |
| *Melicytus* | *obovatus* | C0HKJ7, C0HKJ8 | Red |
| **Oldenlandia* | *affinis* | ADJ17469 | Blue |
| *Orthion* | *oblanceolatum* | Orto A in Burman et al., 2015 | Gray |
| **Palicourea* | *rigida* | AOG74778 | Blue |
| *Rinorea* | *gracilipes* | Rigra A in Burman et al., 2015 | Black |
| *Rinorea* | *lindeniana* | Rili A and Rili B in Burman et al., 2015 | Black |
| *Viola* | *arvensis* | P0C589, P58446, P58447, P58448, P58449, P58451, P58452, P58453, P83835, P83840 | Brown |
| *Viola* | *biflora* | ACZ95469, ACZ95470, ACZ95471, ACZ95472, B1NRQ8, B1NRQ9, B1NRR0, B1NRR1, B1NRR3, P85239, P85240, P85241, P85242, P85244, P85245, P85246, P85525, P85526 | Brown |
| *Viola* | *cotyledon* | P84635, P84636 | Brown |
| *Viola* | *decumbens* | Vide A in Burman et al., 2015 | Brown |
| *Viola* | *hederacea* | P58433, P85232, P85233, P85234 | Brown |
| *Viola* | *inconspicua* | C0HKK1, C0HKK2 | Brown |
| *Viola* | *nivalis* | Vini A in Burman et al. (2015) | Brown |
| *Viola* | *odorata* | 1DF6_A, 1NBJ_A, ACI29316, P58434, P58435, P58436, P58437, P58438, P58439, P58440, P58441, P58442, P58443, P82230, P83836, P83838, P83839, P85177, P85178, P85179, P85180, P85181, P85182, P85183, P85184, P85185, P85186, P85187, P85188, Q2HY54, Q5USN8 | Brown |
| *Viola* | *pubescens* | Gene IDs and expression data are listed in Supplementary Data Sheet 2 | Purple |
| *Viola* | *sp. WO2005118628* | CAJ56194, CAJ56226 | Brown |
| *Viola* | *sp. WO2006041917* | CAN08378 | Brown |
| *Viola* | *tricolor* | AJR10105, ALI30820, ALI30821, ALI30822, ALI30823, ALI30827, ALI30828, ALI30830 | Brown |
| ^*^ denote Rubiaceae species used as outgroups in phylogenetic analyses | | | |

### Table S4

Table S4. ABySS output comparing *V. pubescens* genome assemblies comprised of all four sequenced libraries vs. only two superior libraries that passed FastQC. K-mer sizes 69, 79, and 89 were tested, and a k-mer of 79 was selected for the final assembly.

|  | All Four Libraries (>500 bp) | | | Two Libraries (>500 bp) | | |
| --- | --- | --- | --- | --- | --- | --- |
|  | **k69** | **k79** | **k89** | **k69** | **k79** | **k89** |
| # of scaffolds | 146,872 | 163,177 | 168,108 | 143,732 | 157,722 | 163,792 |
| Total size of scaffolds (bp) | 268,989,943 | 314,048,987 | 346,081,115 | 273,924,499 | 318,370,682 | 312,927,578 |
| Longest scaffold (bp) | 58,077 | 66,219 | 66,239 | 72,290 | 86,685 | 61,315 |
| Number of scaffolds >1kb | 71,091 | 81,676 | 89,402 | 70,814 | 80,885 | 87,740 |
| Number of scaffolds > 10kb | 2,810 | 3,555 | 4,195 | 3,201 | 3,992 | 2,956 |
| Number of scaffolds > 100kb | 0 | 0 | 0 | 0 | 0 | 0 |
| N50 length (bp) | 2,971 | 3,209 | 3,519 | 3,196 | 3,500 | 2,980 |
| Average length of breaks (>25 Ns) between contigs in scaffold | 46 | 46 | 44 | 46 | 45 | 46 |
| Scaffold %N | 0.14 | 0.08 | 0.03 | 0.12 | 0.06 | 0.03 |
| % in scaffolded contigs | 5.7 | 3.8 | 1.5 | 5.4 | 3.2 | 1.6 |
